# Mangiferin supplementation improves serum lipid profiles in overweight patients with hyperlipidemia: a double-blind randomized controlled trial

**DOI:** 10.1038/srep10344

**Published:** 2015-05-19

**Authors:** Lixin Na, Qiao Zhang, Shuo Jiang, Shanshan Du, Wei Zhang, Ying Li, Changhao Sun, Yucun Niu

**Affiliations:** 1 Department of Nutrition and Food Hygiene, Public Health College, Harbin Medical University, Harbin, China

## Abstract

Our previous studies have shown that mangiferin decreased serum triglycerides and free fatty acids (FFAs) by increasing FFAs oxidation in both animal and cell experiments. This study sought to evaluate the effects of mangiferin on serum lipid profiles in overweight patients with hyperlipidemia. Overweight patients with hyperlipidemia (serum triglyceride ≥ 1.70 mmol/L, and total cholesterol ≥ 5.2 mmol/L) were included in this double-blind randomized controlled trial. Participants were randomly allocated to groups, either receiving mangiferin (150 mg/day) or identical placebo for 12 weeks. The lipid profile and serum levels of mangiferin, glucose, L-carnitine, β-hydroxybutyrate, and acetoacetate were determined at baseline and 12 weeks. A total of 97 participants completed the trial. Compared with the placebo control, mangiferin supplementation significantly decreased the serum levels of triglycerides and FFAs, and insulin resistance index. Mangiferin supplementation also significantly increased the serum levels of mangiferin, high-density lipoprotein cholesterol, L-carnitine, β-hydroxybutyrate, and acetoacetate, and increased lipoprotein lipase activity. However, there were no differences in the serum levels of total cholesterol, low-density lipoprotein cholesterol, serum glucose, and insulin between groups. Mangiferin supplementation could improve serum lipid profiles by reducing serum triglycerides and FFAs in overweight patients with hyperlipidemia, partly due to the promotion of FFAs oxidation.

Atherosclerosis and cardiovascular disease are the leading causes of death worldwide^1^,^2^. Hyperlipidemia and elevated free fatty acids (FFAs) are risk factors for these diseases^1^,^3^. An increase of 1 mmol/L in plasma triglyceride levels can lead to a 76% increase in cardiovascular disease risk in women and a 31% increase in men^4^. Elevated plasma triglycerides (TG) and FFA levels can increase the production of atherogenic chylomicron and very-low-density lipoprotein (VLDL) remnants, which can destroy vascular endothelial function and increase arterial stiffness. The possible mechanism of action of these effects is the activation of oxidative stress-responsive transcription factors, producing inflammatory cytokines and increasing the expression of adhesion molecules^5^,^6^. In addition, elevated plasma FFA can also lead directly to insulin resistance, which is strongly correlated with atherosclerosis and cardiovascular disease^7^. Therefore, lowering blood lipids, especially the levels of TG and FFAs, has been considered an effective strategy to reduce overall atherosclerosis and cardiovascular risk, and it was included in The Adult Treatment Panel III^8^,^9^.

Different types of lipid-lowering drugs are widely used, many of which should be used long-term and have various adverse side effects, such as hepatotoxicity, renal toxicity, and neurotoxicity^10^,^11^,^12^. However, it is still a challenge to develop more effective lipid-lowing drugs with fewer adverse side effects. Screening drugs from many phytochemicals may be a possible solution because most phytochemicals are currently considered to be less toxic and have numerous pharmacological functions.

Mangiferin, 1,3,6,7-tetrahydroxyxanthone-C2-β-D-glucoside, is a natural phytochemical present in various plants, including *Anemarrhena asphodeloides, Mangifera indica*, and *Mangifera persiciformis*^13^. It has been shown that mangiferin has many beneficial biological activities, including anti-inflammatory, anti-oxidant, and anti-diabetic effects^14^,^15^,^16^. Moreover, recent animal studies have shown that mangiferin may lower TG and total cholesterol (TC) levels in diabetic rats^17^,^18^. Furthermore, our laboratory has also confirmed that mangiferin had the effects of lowering TG and FFAs in hyperlipidemic hamsters, rats, and hepG2 cell models, which may have been achieved by modulation of the key enzyme expression involved in inhibiting lipogenesis and promoting fatty acid oxidation in the liver^19^,^20^. However, the effect of mangiferin on blood lipids in overweight patients with hyperlipidemia remains unclear.

Based on the lipid-lowering effects of mangiferin in animal and cell experiments, we conducted this double-blind randomized clinical trial to evaluate the effects of mangiferin on blood lipid profiles in overweight patients with hyperlipidemia.

## Results

### Study participants

**Figure 1:**
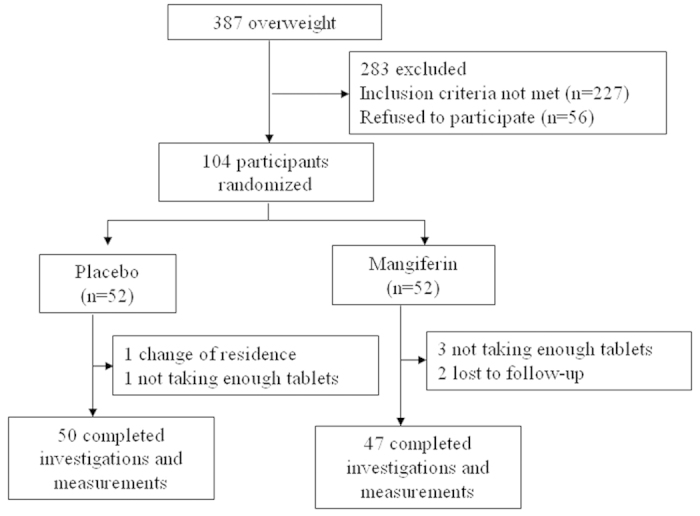
Study design and the flow of participants.

Among the 104 eligible individuals who participated in the study at baseline, 97 completed the trial and took all the tablets. Two individuals in the placebo group were excluded from the analysis because they changed their place of residence (n = 1) or did not take the suggested quantity of tablets (n = 1). Five individuals did not complete the study in the mangiferin supplementation group because they did not take the suggested quantity of tablets (n = 3) or were lost to follow-up (n = 2) (Fig. 1). There were no significant differences in the overall compliance rates between the two groups (*P* = 0.257).

### Characteristics of the participants

At baseline, there were no significant differences between the placebo and mangiferin supplementation groups with respect to age, gender, body mass index (BMI), and blood pressure. There was also no difference in the lipid-lowering statin drugs taken between the two groups (Table 1). Dietary nutrient intakes in both groups did not differ significantly between the two groups at either baseline or the end of the intervention, suggesting that mangiferin did not suppress appetite at the dose used (Table 2).

### Serum lipids, serum glucose, and other metabolic variables

There were no significant differences in any variables between the two groups at baseline. After 12 weeks of the intervention, there was a significant increase in serum mangiferin levels and a significant decrease in serum TG and FFA levels in the mangiferin supplementation group compared with the control group. Furthermore, mangiferin significantly increased the levels of serum high-density lipoprotein (HDL), L-carnitine, β-hydroxybutyrate, and acetoacetate levels. Although there were no differences in serum glucose and insulin levels between the control and mangiferin groups, mangiferin supplementation significantly decreased the insulin resistance index (HOMA-IR) compared with that of the placebo group. No significant changes in serum TC and low-density lipoprotein (LDL) levels were found after the end of the mangiferin intervention (Table 3).

**Figure 2:**
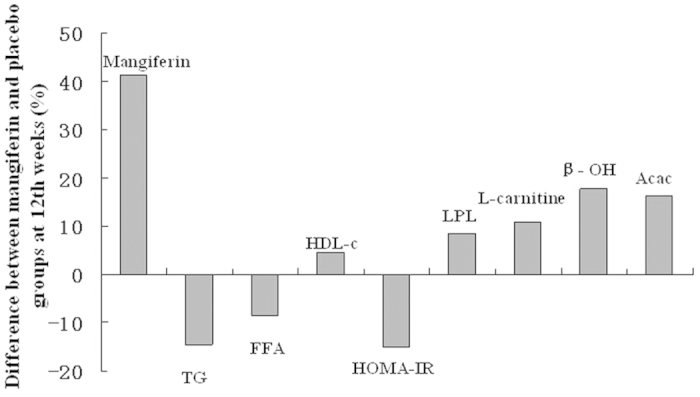
Differences between the mangiferin supplementation and placebo groups with respect to significant variables at the 12th week, adjusted for baseline values, serum mangiferin, age, gender, BMI, blood pressure, dietary intake, hyperlipidemia duration, and drug treatment.

Figure 2 shows the percentage differences in serum profiles between the mangiferin supplementation and placebo groups at the 12th week, adjusted for baseline values, serum mangiferin, age, gender, BMI, blood pressure, physical activity, hyperlipidemia duration, and drug treatment determined by an analysis of covariance (ANCOVA). Compared with placebo, mangiferin supplementation significantly decreased the TG, FFA, and HOMA-IR by 14.4%, 8.4%, and 8.4%, respectively, and increased serum mangiferin, HDL, LPL, L-carnitine, β-hydroxybutyrate, and acetoacetate by 41.2%, 4.65%, 15.1%, 11.0%, 17.1%, and 16.5%, respectively.

### Serum FFA profile

Total serum fatty acids, total serum saturated fatty acids (SFA), total serum mono-unsaturated fatty acids (MUFA), total serum poly-unsaturated fatty acids (PUFA), total serum n-3 fatty acids, and total serum n-6 fatty acids were significantly decreased in the mangiferin supplementation group compared with the controls. Further analysis of the serum FFA profiles showed that the contents of most fatty acids were also significantly decreased in the mangiferin supplementation group; only the contents of C14:0 and γ-C18:3 were not changed by mangiferin intervention (Table 4).

### Hematologic measures and liver and kidney variables

Mangiferin supplementation had no significant effects on red blood cell, white blood cell, and platelet counts or hemoglobin, total protein, albumin, urea nitrogen, creatinine, aspartate aminotransferase (AST), and alanine transaminase (ALT) levels compared with the control group (Table 5).

## Discussion

This randomized, double-blind, placebo-controlled trial revealed that mangiferin supplementation for 12 weeks significantly decreased the serum TG, FFA, and HOMA-IR levels and increased the serum mangiferin, HDL, and LPL levels in overweight patients with hyperlipidemia. In addition, levels of L-carnitine, β-hydroxybutyrate, and acetoacetate, which are used to diagnose fatty acid metabolism, were also increased by mangiferin. These results suggested that mangiferin supplementation could improve serum lipid profiles through the lowering of serum TG and FFA levels by promoting FFA oxidation.

Our previous study showed that one dose of oral mangiferin (900 mg) in humans did not produce any side effects or changes in clinical symptoms and blood biochemical variables^21^. As there have been no reports regarding mangiferin intervention for a long period of time in any human study, we selected a relatively low and safer dose of mangiferin (150 mg/d), which is 1/2 the 300 mg/d dose applied in rats. We reported that the lowest effective dose of mangiferin was 50 mg/kg body weight (BW) in rats^19^,^20^. The conversion coefficient from rats to humans is 6 according to the body surface area estimation method^22^. Therefore, the dose of 300 mg/d mangiferin in humans was calculated by 50 mg/kg BW ^^*^^ 6. In addition, we did not observe any side effects or changes in liver enzymes or kidney variables in the participants after the 12-week intervention.

Our previous studies showed that mangiferin could lower serum TG and FFA by regulating the key enzymes of FFA synthesis and oxidation in liver and muscle tissues in rats or HepG2 cells. This study aimed to investigate the effects of mangiferin on lowering serum TG and FFA in human intervention trials. The results showed that mangiferin supplementation significantly decreased serum TG levels in overweight patients with hyperlipidemia. The mechanism involved in lowering serum TG is either the inhibition of TG synthesis or acceleration of TG decomposition^23^,^24^,^25^, and the catabolism of TG was mainly investigated in this study. Serum TG is first hydrolyzed, which liberates FFA during TG catabolism^26^. Lipoprotein lipase (LPL) is the key enzyme that hydrolyzes circulating TG, delivering FFAs to peripheral tissues for utilization and storage^27^,^28^. In our study, serum TG was significantly decreased in the mangiferin supplementation group and serum LPL activity was significantly increased, which suggested that mangiferin at least partly increased TG hydrolysis by LPL, and the decrease in serum TG was due to the increase in serum TG catabolism. In accordance with our findings, another recent animal study demonstrated that mangiferin could upregulate proteins important for mitochondrial bioenergetics and downregulate proteins controlling de novo lipogenesis, which could enable mangiferin to enhance energy expenditure and inhibit lipogenesis^29^. Apontes P *et al.* reported that mangiferin exerts its effects of lowering serum TG and improving glucose and insulin profiles by stimulating carbohydrate oxidation^30^.

Approximately 10% of serum FFAs are derived from the hydrolysis of serum TG, and serum FFA levels should increase with the increase in TG hydrolysis^31^,^32^. However, the total FFA level was significantly decreased in the mangiferin supplementation group compared with the control group in our study. Furthermore, the effects of mangiferin supplementation on serum FFA profiles were observed in this study, and the results showed that SFA, MUFA, PUFA, total serum n-3 fatty acids, and total serum n-6 fatty acids in serum FFA profiles were significantly decreased in the mangiferin supplementation group, and only C14:0 and γ-C18:3 had no statistically significant change after the intervention with mangiferin for 12 weeks. Therefore, we speculated that mangiferin decreased serum TG by the reduction of serum total FFA. However, there was no significant effect of mangiferin supplementation on the chain lengths and degree of unsaturation of FFA.

The major organ for FFA metabolism is the liver at rest and the muscle during activity^33^. FFA enters these organs, where it can either be oxidized in the mitochondria to form ATP or esterified to produce TG for storage or incorporation into VLDL particles^34^. L-carnitine and carnitine palmitoyltransferase (CPT) are essential for the translocation of long-chain fatty acids that have entered the mitochondria, and their quantities have been used for the testing of fatty acid metabolism^35^. Serum L-carnitine levels increase when β-oxidation rates are in excess of the rate of complete oxidation to CO_2_ through the tricarboxylic acid (TCA) cycle^36^,^37^. In our study, the serum L-carnitine level was significantly increased after mangiferin supplementation for 12 weeks, which indicated that mangiferin increased FFA oxidation by promoting the movement of FFA into mitochondria for oxidation by the carnitine carrier.

β-Hydroxybutyrate and acetoacetate may also help us to understand the above data. β-Hydroxybutyrate and acetoacetate (ketone body) are the main alternative energy substrates to glucose, and they are mainly generated by the oxidation of FFA that are transported into the blood and utilized as a fuel for other cells; therefore, they should also be elevated in parallel with the increase in FFA oxidation^38^,^39^,^40^. However, β-hydroxybutyrate and acetoacetate were significantly increased in the mangiferin supplementation group in our study. In addition, we demonstrated that mangiferin could increase serum β-hydroxybutyrate in hyperlipidemic rats^20^. Therefore, we concluded that the increased β-hydroxybutyrate and acetoacetate levels induced by mangiferin may have originated from the increase in FFA oxidation in the mitochondria.

In conclusion, our results suggest that mangiferin can decrease the levels of serum TG and FFAs. It can also promote FFA oxidation by increasing serum L-carnitine, β-hydroxybutyrate, and acetoacetate levels in overweight patients with hyperlipidemia. However, further studies are needed to evaluate and quantify the rate of FFA oxidation in overweight patients with hyperlipidemia treated by mangiferin.

## Methods

### Participants

Study participants with established hyperlipidemia were recruited from five communities in Harbin, China. The primary selection criteria included the following: age between 35 and 65 years; BMI ≥ 24.0; no cardiovascular disease, diabetes mellitus, or liver/renal disease; and a history of hyperlipidemia based on a print-out dated within half a year showing a plasma TG concentration ≥ 1.70 mmol/L and plasma TC concentration ≥ 5.20 mmol/L. Participants were excluded if they had taken traditional Chinese medicine or lipid-lowing drugs, except for statins. Based on the examination of 387 overweight subjects, we recruited a total of 104 eligible overweight patients with hyperlipidemia who agreed to participate in this study. All participants provided written informed consent to participate in this study, which was approved by the Human Ethics Committee of the Harbin Medical University.

### Mangiferin intervention

Before the study was conducted, we treated rats and hamsters with mangiferin for 6 weeks. We observed significant decreases in blood lipid levels caused by mangiferin. We selected a relatively lower and safer dose of mangiferin (150 mg/d) for the study participants, which was 1/2 the dose of 300 mg/d used in our previous animal studies^19^ and was calculated based on the specific surface area according to the weight of the average human (60 kg). Mangiferin powder (purity 98%) was purchased (Xi An, China) and extracted from *Anemarrhena asphodeloides Bunge*. Mangiferin was incorporated into tablets using maize starch by Harbin Pharmaceutical Group 6th Pharm Factory, with each tablet containing 150 mg mangiferin. An identical placebo was produced, which contained only maize starch. Participants were asked to take 1 tablet each day.

### Study design

This 12-week, double-blind, placebo-controlled study was registered at Current Controlled Trials (http://www.chictr.org, registration number: ChiCTR-TRC-13003962). The participants were stratified by serum TG levels and randomly assigned to take either placebo or mangiferin (150 mg/d). Participants were instructed not to change their usual diets, physical activity levels, or medications throughout the study. The participants were assigned to either supplement A or B according to a computer-generated blocked randomization schedule. The study coordinator assigned the participants to the supplement specified by the schedule in chronological order as the participants enrolled. Assignment into each study group remained concealed until participant enrollment was completed. A safety monitor who was otherwise uninvolved with the study held the key to the blinded assignments in a sealed envelope until all results were obtained and analyzed.

Compliance of the study participants was assessed by conducting scheduled telephone interviews and by counting the remaining tablets every two weeks. The study was approved by the ethics committee of Harbin Medical University and was conducted in accordance with the Declaration of Helsinki. Written informed consent was obtained from all participants.

### Demographic characteristics

Demographic data were collected at baseline using a standardized questionnaire. Information collected included age, gender, physical activity, current smoking habits, hyperlipidemia duration, and medications. Physical activity level was assessed using a physical activity questionnaire and calculated using a formula from the American Institute of Medicine^41^. At baseline and the 12th week, dietary intakes were measured with a validated semi-quantitative food-frequency questionnaire (FFQ). Energy and nutrient intakes were calculated using the Food Nutrition Calculator (V1.60; Shixinghengxun, Beijing, China).

### Anthropometric and blood chemistry measurements

Anthropometric indices were measured by well-trained examiners with participants wearing light, thin clothing and no shoes. Body weight and height were measured to the nearest 0.1 kg and 0.1 cm, respectively. All participants were instructed to fast for 10 h, and fasting venous blood samples were collected from the elbow in the morning both at baseline and at 12 weeks. Serum mangiferin was determined by high-performance liquid chromatography-mass spectrometry (HPLC-MS) according to our established method^21^. Serum TC, TG, HDL, LDL, glucose, β-hydroxybutyrate, acetoacetate, AST, ALT, total protein, albumin, and blood urea nitrogen (BUN) were determined using a ROCHE Modular P800 Automatic Biochemical Analyzer (Roche Diagnostics, Mannheim, Germany). Serum insulin concentration was measured by a ROCHE Elecsys 2010 Chemiluminescence Immune Analyzer (Roche Diagnostics). Serum carnitine level was determined by enzymatic colorimetric methods with commercial kits (Bangjing, Shanghai, China). Serum LPL concentration was measured by an enzyme-linked immunosorbent assay (ELISA) using commercially available ELISA Kits (R&D System, USA). Serum FFA were determined by Gas Chromatography-Mass Spectrometer (TRACE GC/PolarisQ MS, Thermo Finnigan, USA) as described previously by our laboratory^42^. The blood cell count and hemoglobin concentration were determined using a SYSMEX-SF-3000 Automatic Hematology Analyzer (Sysmex Corporation, Kobe, Japan). The insulin resistance index (HOMA-IR) was calculated using the following formula: fasting insulin (μU/ml) × fasting blood glucose (mmol/l) / 22.5^43^.

### Statistical analysis

Data are presented as the mean ± SD or percentage. The χ2 test was used to compare categorical variables. Mean levels of continuous study variables at baseline and the 12th week were compared between the two groups by an analysis of covariance (ANCOVA). Paired-samples t tests were used to evaluate the changes in outcome variables before and after the intervention in each group. The ANCOVA was adjusted for baseline values of serum mangiferin, age, gender, BMI, blood pressure, dietary intake, hyperlipidemia duration, and drug treatment. The percentage differences between the mangiferin and placebo groups in outcome variables were calculated using covariate-adjusted values, such as 100 × (treatment group-placebo group)/placebo group, for each study variable. Statistical analyses were carried out using SPSS (version 13.01S, Beijing Stats Data Mining Co. Ltd, Beijing, China). All *P* values were two-tailed, and *P* < 0.05 was considered to indicate statistical significance.

## Author Contributions

LX.N., Q.Z., Y.L., CH.S. and YC.N. conceived and designed the experiments. Q.Z., S.J., SS.D. and W.Z. performed the experiments and analyzed the data. LX.N. and YC.N. wrote the main manuscript text. Q.Z. and S.J. prepared all figures and tables. All authors reviewed the manuscript.

## Additional Information

**How to cite this article**: Na, L. *et al.* Mangiferin supplementation improves serum lipid profiles in overweight patients with hyperlipidemia: a double-blind randomized controlled trial. *Sci. Rep.* **5**, 10344; doi: 10.1038/srep10344 (2015).

## Figures and Tables

**Table 1:** Characteristics of participants at baseline.

**Variable**	**Placebo group (n** = **50)**	**Mangiferin group (n** = **47)**
Age (years)	52.1 ± 7.2	52.6 ± 7.3
Male, n (%)	27 (51.92)	26 (55.32)
BMI (kg/m^2^)	26.7 ± 4.8	27.1 ± 4.2
Systolic BP (mm Hg)	140.18 ± 12.88	139.02 ± 10.86
Diastolic BP (mm Hg)	84.42 ± 7.96	83.96 ± 6.34
Current smokers, n (%)	3 (6.0)	2 (4.3)
Physical activity level	1.81 ± 0.37	1.78 ± 0.39
Hyperlipidemia duration (years)	5.9 ± 1.4	5.6 ± 1.4
Statin medications n (%)	8 (16.0)	7 (14.8)

**Table 2:** Main daily intake of nutrients by the subjects at baseline and after the intervention

	**Placebo group (n** = **50)**		**Mangiferin group (n** = **47)**		**P**
	**Baseline**	**End of intervention**	**Baseline**	**End of intervention**	
Energy (kcal/d)	2701.2 ± 412.3	2735.2 ± 502.8	2693.6 ± 479.2	2719.6 ± 493.5	0.842
Protein (g/d)	88.3 ± 22.4	85.4 ± 26.2	78.5 ± 20.6	89.1 ± 24.2	0.472
Fat (g/d)	95.6 ± 26.4	98.5 ± 25.3	97.8 ± 27.6	99.8 ± 27.1	0.807
Carbohydrates (g/d)	395.8 ± 105.2	401.4 ± 116.7	399.3 ± 94.6	391.2 ± 108.1	0.657

*P* values were calculated for the differences between the placebo and mangiferin groups at 12 weeks. The effects of the mangiferin intervention on these variables were analyzed by ANCOVA, with the baseline values of serum mangiferin, age, gender, BMI, blood pressure, physical activity, hyperlipidemia duration, and drug treatment used as covariates.

**Table 3:** Clinical variables at baseline and at 12 weeks in the mangiferin supplementation and placebo groups.

**Variable**	**Placebo group (n** = **50)**		**Mangiferin group (n** = **47)**		***P***
	**Baseline**	**End of intervention**	**Baseline**	**End of intervention**	
Mangiferin (ng/mL)	9.11 ± 0.75	10.02 ± 0.89	9.97 ± 1.03	14.15 ± 2.98	0.001
TC (mmol/L)	6.66 ± 0.74	6.55 ± 0.86	6.62 ± 0.67	6.32 ± 0.65	0.132
TG (mmol/L)	2.06 ± 0.26	2.01 ± 0.27	2.05 ± 0.23	1.72 ± 0.19	0.001
HDL cholesterol (mmol/L)	1.27 ± 0.12	1.29 ± 0.11	1.26 ± 0.13	1.35 ± 0.15	0.029
LDL cholesterol (mmol/L)	2.98 ± 0.38	2.93 ± 0.35	3.02 ± 0.45	2.85 ± 0.41	0.303
LPL (U/L)	681.3 ± 132.5	695.1 ± 147.6	703.4 ± 127.4	753.5 ± 122.7	0.037
Glucose (mmol/L)	5.92 ± 1.63	5.89 ± 1.61	6.01 ± 1.67	5.52 ± 1.52	0.248
Insulin (μU/mL)	9.12 ± 4.62	9.34 ± 4.96	9.21 ± 4.75	8.42 ± 4.13	0.325
HOMA-IR	2.61 ± 1.06	2.65 ± 1.03	2.69 ± 1.12	2.25 ± 1.01	0.046
L-carnitine (μmol/L)	24.32 ± 5.64	24.92 ± 6.05	23.14 ± 5.26	27.67 ± 6.12	0.028
β-hydroxybutyrate (μmol/L)	85.15 ± 14.91	93.06 ± 14.31	89.14 ± 16.87	109.51 ± 16.44	0.001
Acetoacetate (μmol/L)	45.51 ± 15.72	43.16 ± 15.84	45.32 ± 17.37	50.27 ± 15.66	0.028

*P* values were calculated for the differences between the placebo and mangiferin groups at 12 weeks. The effects of the mangiferin intervention on these variables were analyzed by ANCOVA, with the baseline values of serum mangiferin, age, gender, BMI, blood pressure, dietary intake, hyperlipidemia duration, and drug treatment used as covariates.

**Table 4:** Fasting plasma fatty acid profiles in serum before and after mangiferin supplementation.

**Fatty acids (μmol/L)**	**Placebo (n** = **50)**		**Mangiferin (n** = **47)**		***P***
	**Baseline**	**End of intervention**	**Baseline**	**End of intervention**	
C14:0	18.7 ± 8.2	17.6 ± 6.9	19.2 ± 7.3	15.8 ± 5.6	0.155
C16:0	1812.4 ± 91.3	1842.1 ± 99.8	1821.9 ± 83.5	1745.7 ± 87.1	<0.001
C16:1	155.3 ± 16.7	149.6 ± 15.3	161.1 ± 20.4	140.9 ± 18.9	0.013
C18:0	682.4 ± 46.3	667.1 ± 38.7	679.6 ± 42.9	637.2 ± 41.4	<0.001
C18:1	931.4 ± 50.5	917.6 ± 59.1	943.4 ± 55.4	893.2 ± 48.9	0.026
C18:2	941.5 ± 55.1	877.0 ± 53.2	977.7 ± 62.5	783.7 ± 54.4	<0.001
γ-C18:3	29.3 ± 4.3	29.1 ± 5.4	29.7 ± 5.2	27.9 ± 5.1	0.256
C18:3	58.5 ± 6.3	55.5 ± 4.9	58.2 ± 6.2	53.3 ± 4.6	0.023
C20:2	52.8 ± 3.4	51.4 ± 3.4	51.6 ± 2.8	49.8 ± 3.9	0.031
C20:4	640.7 ± 50.5	662.2 ± 47.9	670.3 ± 47.9	606.2 ± 43.0	<0.001
C20:5	41.7 ± 3.5	42.5 ± 3.1	43.5 ± 3.2	40.4 ± 3.6	0.002
C22:5	99.4 ± 8.7	103.5 ± 9.2	104.8 ± 10.0	94.3 ± 8.1	<0.001
C22:6	146.9 ± 18.5	152.7 ± 17.1	150.2 ± 18.1	143.2 ± 19.5	0.011
C24:0	10.1 ± 2.5	10.4 ± 2.6	10.5 ± 2.1	9.2 ± 2.4	0.018
Total SFFA	2523.6 ± 133.5	2537.2 ± 151.2	2531.2 ± 139.4	2407.9 ± 138.6	<0.001
Total MUFFA	1086.7 ± 75.3	1067.2 ± 60.4	1104.5 ± 68.5	1034.1 ± 79.3	0.022
Total PUFFA	2010.8 ± 100.5	1973.9 ± 139.4	2086.0 ± 116.9	1798.8 ± 140.8	<0.001
Total n-3 FFA	346.5 ± 41.2	354.2 ± 50.2	356.7 ± 37.7	331.4 ± 44.4	0.020
Total n-6 FFA	1670.0 ± 98.6	1619.7 ± 71.5	1729.3 ± 59.7	1467.6 ± 77.9	<0.001
Total FFA	5621.1 ± 183.4	5721.7 ± 225.8	5578.3 ± 212.2	5240.8 ± 196.7	<0.001

*P* values were calculated for the differences between the placebo and mangiferin groups at 12 weeks. The effects of the mangiferin intervention on these variables were analyzed by ANCOVA, with the baseline values of serum mangiferin, age, gender, BMI, blood pressure, dietary intake, hyperlipidemia duration, and drug treatment used as covariates.

**Table 5:** Blood chemistry results, hematologic measures, and liver enzyme values for the hyperlipidemia patients

	**Placebo (n** = **50)**		**Mangiferin (n** = **47)**		***P***
	**Baseline**	**End of intervention**	**Baseline**	**End of intervention**	
Hemoglobin (g/L)	133.8 ± 10.2	134.4 ± 8.1	135.2 ± 11.0	132.9 ± 11.6	0.455
Red blood cells (×10^12^ /L)	4.58 ± 0.47	4.57 ± 0.41	4.62 ± 0.38	4.69 ± 0.32	0.151
White blood cells (×10^12^ /L)	6.63 ± 1.48	6.61 ± 1.77	6.59 ± 1.61	6.62 ± 1.44	0.975
Platelets (×10^9^ ^/^L)	205.7 ± 31.5	203.6 ± 32.8	211.2 ± 35.6	211.3 ± 33.4	0.248
Total protein (g/L)	74.7 ± 4.9	76.1 ± 5.8	75.7 ± 5.5	74.7 ± 5.3	0.211
Albumin (g/L)	41.7 ± 3.6	42.1 ± 3.8	42.4 ± 3.0	41.7 ± 3.6	0.590
Aspartate transaminase (U/L)	27.7 ± 7.6	27.2 ± 7.8	25.7 ± 8.7	25.7 ± 6.4	0.296
Alanine transaminase (U/L)	14.4 ± 5.5	14.1 ± 5.9	14.4 ± 5.1	14.4 ± 5.6	0.795
Urea nitrogen (mmol/L)	5.2 ± 0.8	5.2 ± 0.8	5.2 ± 0.8	5.0 ± 0.7	0.186
Creatinine (μmol/L)	70.5 ± 9.5	70.6 ± 11.7	71.0 ± 10.7	71.6 ± 9.6	0.641

*P* values were calculated for the differences between the placebo and mangiferin groups at 12 weeks. The effects of the mangiferin intervention on these variables were analyzed by ANCOVA, with the baseline values of serum mangiferin, age, gender, BMI, blood pressure, dietary intake, hyperlipidemia duration, and drug treatment used as covariates.
